# News and views (9&10): 2022 AAPPS-APCTP C.N. Yang Award; GR23 Report; 48th & 49th AAPPS Council Meetings

**DOI:** 10.1007/s43673-022-00061-4

**Published:** 2022-09-26

**Authors:** AAPPS Bulletin

**Affiliations:** Association of Asia Pacific Physical Societies, Pohang, Korea

## The 2022 AAPPS-APCTP C.N. Yang Award by Rajdeep Singh Rawat (The Chair of the C.N. Yang Award Committee)

We are very pleased to announce the three outstanding young scholars who are receiving the 2022 AAPPS-APCTP C.N. Yang Award (C.N. Yang Award): Guancong Ma (Hong Kong Baptist University), Jianwei Wang (Peking University), and Meng-Ru Wu (Academia Sinica). This year, a total of 31 nominations were received; the nominees hailed from 11 different countries and their respective areas of research were represented in 10 distinct sessions of the Asia Pacific Physics Conference (APPC-15) (see Table 1). This year, 27 nominations were from AAPPS member societies and AAPPS divisions, while the remaining four nominations were individual nominations. All the nominees from the AAPPS member societies and AAPPS divisions and from the high-profile experts were excellent, making the selection process highly challenging.

**Table 1:** The nationalities and the sessions of the 2022 C.N. Yang Award nominees

	No. of candidates
**Nationality**	
Japan	8
China/Beijing	7
Korea	6
India	2
Hong Kong	2
China/Taipei	1
Kazakhstan	1
Singapore	1
Vietnam	1
Poland	1
Canada	1
**Total**	**31**
**Sessions (in APPC15-2022)**	
A. Applied Physics	1
B. Astrophysics, Cosmology, and Gravitation	6
C. Atomic and Molecular Physics	1
E. Condensed Matter Physics	9
G. Nuclear Physics	3
I. Particles and Fields	1
K. Plasma Physics	1
L. Quantum Information	3
M. Semiconductor Physics	4
N. Statistical Physics	2
**Total**	**31**

The C.N. Yang Award was established to honor and encourage young researchers with prominent research achievements and to promote the next generation’s leading scholars in physics in the Asia Pacific region. This award had been presented during the Asia Pacific Physics Conference (APPC), which has been held approximately every three years. Notably, starting in 2019, the Association of Asia Pacific Physical Societies (AAPPS) and the Asia Pacific Center for Theoretical Physics (APCTP) jointly established the AAPPS-APCTP Chen-Ning Yang Award (C.N. Yang Award) in order to make it an annual event. The C.N. Yang Award Committee consists of members from the AAPPS Council, AAPPS Divisions, and renowned scholars recommended by APCTP. This year, the awards will be presented at APPC15 (https://www.appc15.org/ ), which will be held in a hybrid format from August 21 to 26, 2022, in Seoul, Korea.

The selection process is a multi-step and highly challenging process, requiring careful and detailed evaluations at each stage. The originality of the candidates’ works, novelty and impact of their research, and their likely prospects were some of the important areas of consideration. The citations and the briefings for the C.N. Yang 2022 awardees are listed below.

**Guancong Ma, Hong Kong Baptist University**


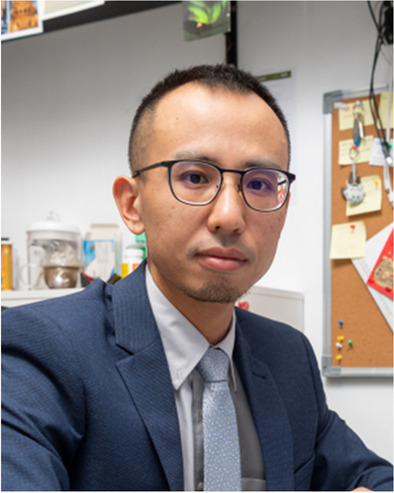

“For his pioneering investigations of novel Hermitian and non-Hermitian topological phases in wave systems”

Dr. Guancong Ma obtained his PhD degree in 2012 from the Hong Kong University of Science and Technology and currently is an associate professor in the Department of Physics at Hong Kong Baptist University. Dr. Ma’s main research interests lie in the realms of topological physics, non-Hermitian physics in classical-wave platforms, and metamaterials. He studies topological physics using classical waves. His works have not only exemplified the universality of topology as a foundation in physics but have also vitalized the study of classical waves by bringing new tools for wave manipulations. For instance, his research opens a new frontier for topological physics by linking it to non-Hermitian systems — a new physical formalism that describes open systems. Singularities called “exceptional points” (EPs) can emerge in the parameter space of non-Hermitian systems. Dr. Ma was the first to experimentally realize higher-order exceptional points.

Dr. Ma pioneered and made continuous contributions to topological wave research. He played a key role in the first realization of a topological transition in sound and subsequently led a series of works that contributed to the vibrant development of topological acoustics. His work on topological physics contributed to the new realm of non-Abelian physics, which is a cornerstone of quantum-logic operations that are described by unitary matrices. He has also made impactful contributions to acoustic metamaterials, demonstrating the excellent sound-proofing capabilities of metamaterials. Dr. Ma later used absorptive metamaterials to break the focusing diffraction limit in three dimensions. Recently, he blurred the boundary between solids and fluids by first demonstrating fluid-like elasticity in a solid-based mechanical metamaterial and conversely realized transversality and spin-orbit interactions in a fluid-acoustic system.

**Jianwei Wang, Peking University**


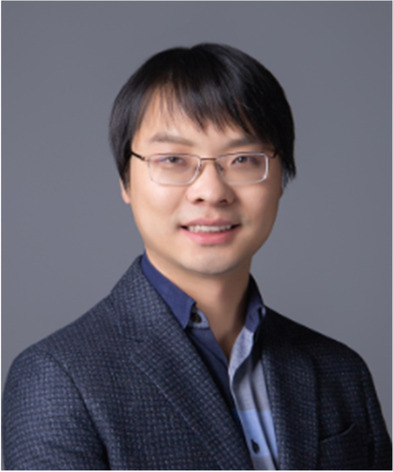

“For his contribution in integrated photonic quantum information science and technologies”

Dr. Jianwei Wang is an assistant professor in the Physics School of Peking University. He received both a BS (2008) and MS degree (2011) in optical engineering at Zhejiang University (China) and then obtained his PhD (2016) in physics at the University of Bristol (UK). Dr. Wang is currently leading a group called the “Integrated Quantum Optics Lab” in the Physics School of Peking University. His research focuses on quantum information science and technologies with photons. He has developed large-scale integrated quantum photonic circuits and devices in silicon and has developed versatile technologies to understand quantum foundations and to explore applications of quantum information theory in communications, simulations, and computing. Dr. Wang realized the world’s first integrated optical quantum chip with over 500 components, which significantly pushed the development of the field. He has made significant contributions to on-chip generation, manipulation, and measurement of complex entanglement structures, including multiphoton entanglement and multidimensional entanglement. His team recently demonstrated the first topologically protected Einstein-Podolsky-Rosen entanglement sources, showing the possibilities of making robust quantum photonic devices. His group realized the first on-chip Gaussian sampling quantum computer that can be reprogrammed to implement quantum simulation of molecular dynamics. Dr. Wang realized the first photonic quantum simulator that can learn the Hamiltonian of quantum systems using hybrid quantum-classical algorithms. His group recently demonstrated the first programmable quantum processor with qudits and also on-chip implementation of eight-qubit cluster-state quantum computing and the benchmarking of error correction with photons stepping toward universal quantum computation. In addition, his group demonstrated the first chip-to-chip entanglement distribution and quantum teleportation, showing a pathway to a chip-based entanglement quantum network.

**Meng-Ru Wu, Academia Sinica**


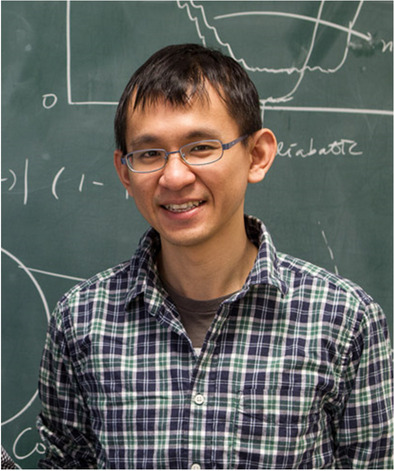

“For his contributions in understanding the origin of heavy elements and in the theoretical development of collective neutrino flavor oscillations in neutron star mergers and in supernovae”

Dr. Meng-Ru Wu is currently an associate professor at the Institute of Physics, Academia Sinica, Taipei. To understand the origin of heavy elements is one of the open problems in physics and astrophysics. The multi-messenger observations of GW 170817 provide evidence for heavy elements being derived from the merger of neutron stars. Dr. Wu, with his collaborators, studied theoretical topics related to r-process nucleosynthesis and neutrino flavor conversions in mergers and supernovae. His work showed that r-process nucleosynthesis in outflows ejected viscously from post-merger black-hole accretion disk systems can robustly produce elemental abundance distributions that match well with what is inferred from the solar system and metal-poor star observations. For the theoretical modeling of kilonova lightcurves powered by the nuclear energy released from the decay of unstable nuclei made in the r-process, Dr. Wu and his collaborators found that the thermalization of particle species produced by different decay channels can largely affect the observable. He performed neutrino detection analysis and nucleosynthesis calculations to predict the elemental yields. For neutrino flavor conversions, he showed that neutron star merger remnants generally host favorable conditions for novel fast neutrino flavor conversions to occur within a length scale of centimeters.

Dr. Wu’s research work offers a new way to potentially understand the exact nucleosynthesis yields of very heavy nuclei with upcoming observations. In another important work, Dr. Wu showed old kilonova remnants residing inside our Milky Way can produce gamma-ray lines from the decay of a few long-lived r-process nuclei, which may be detectable by next-generation gamma-ray telescopes and could yield clues to key questions related to the merger r-process and the property of neutron star binaries.

## Report on the General Relativity and Gravitation (GR23) by Zong-Kuan Guo (DACG)

The 23rd International Conference on General Relativity and Gravitation (GR23) and the 2022 annual meeting of the Division of Gravitation and Relativistic Astrophysics, Chinese Physical Society, were successfully held in Liyang, China, from July 3 to 8, 2022. GR23 was hosted by the Institute of Theoretical Physics, Chinese Academy of Sciences (CAS). Organizational support was provided by Beijing Normal University, Hangzhou Institute for Advanced Study of UCAS, Huazhong University of Science and Technology, Hunan Normal University, Jilin University, Lanzhou University, Shanghai University, Sun Yat-sen University, Tianjin University, University of CAS, Yangzhou University, Zhejiang University of Technology, Universe (ISSN 2218-1997, Published by MDPI), the Division of Gravitation and Relativistic Astrophysics of the Chinese Physical Society, the International Union of Pure and Applied Physics (IUPAP), the Chinese Academy of Sciences, and the National Natural Science Foundation of China. Due to COVID-19, GR23 was held as a hybrid conference, with online and onsite components.

GR23 is the latest in the series of triennial international conferences held under the auspices of the International Society on General Relativity and Gravitation (ISFRG). The conference series constitutes the principal international meetings for scientists working in all areas of relativity and gravitation. GR23 was attended, in terms of both online and onsite participants, by more than 1100 scholars from 52 countries and regions in the world. Seventeen plenary speakers and 386 parallel speakers discussed aspects of the recent progress that has been made in relativity and gravitation. Professor Adam Riess from Johns Hopkins University, who was awarded the 2011 Novel Prize in physics, gave a public lecture titled “Surprises from the Expansion of the Universe.”

Professor Nils Andersson, the ISGRG President, chaired the awards ceremony. There was a brief presentation of the two 2022 thesis prizes, i.e., the Jürgen Ehlers Prize in areas of mathematical and numerical general relativity, and the Bergmann-Wheeler Prize, which encompasses a broad area that includes all approaches to quantum gravity. This presentation was followed by three talks from the IUPAP Early Career Award winners of 2020–2022, who are Davide Gerosa from the University of Milano-Bicocca, Christopher Berry from the University of Glasgow, and Katerina Chatziioannou from Caltech.

At the ISGRG General Assembly, the new ISGRG Fellows were announced. The 2022 ISGRG Fellows are Beverly Berger, Robert Caldwell, Roberto Emparan, John Friedman, Malcolm Maccallum, Mukund Rangamani, Harvey Reall, Luciano Rezzolla, Sheila Rowan, Susan Scott, and Matt Visser. Moreover, it was announced that GR24 will be hosted by the University of Glasgow, UK.

**Fig. 1:**
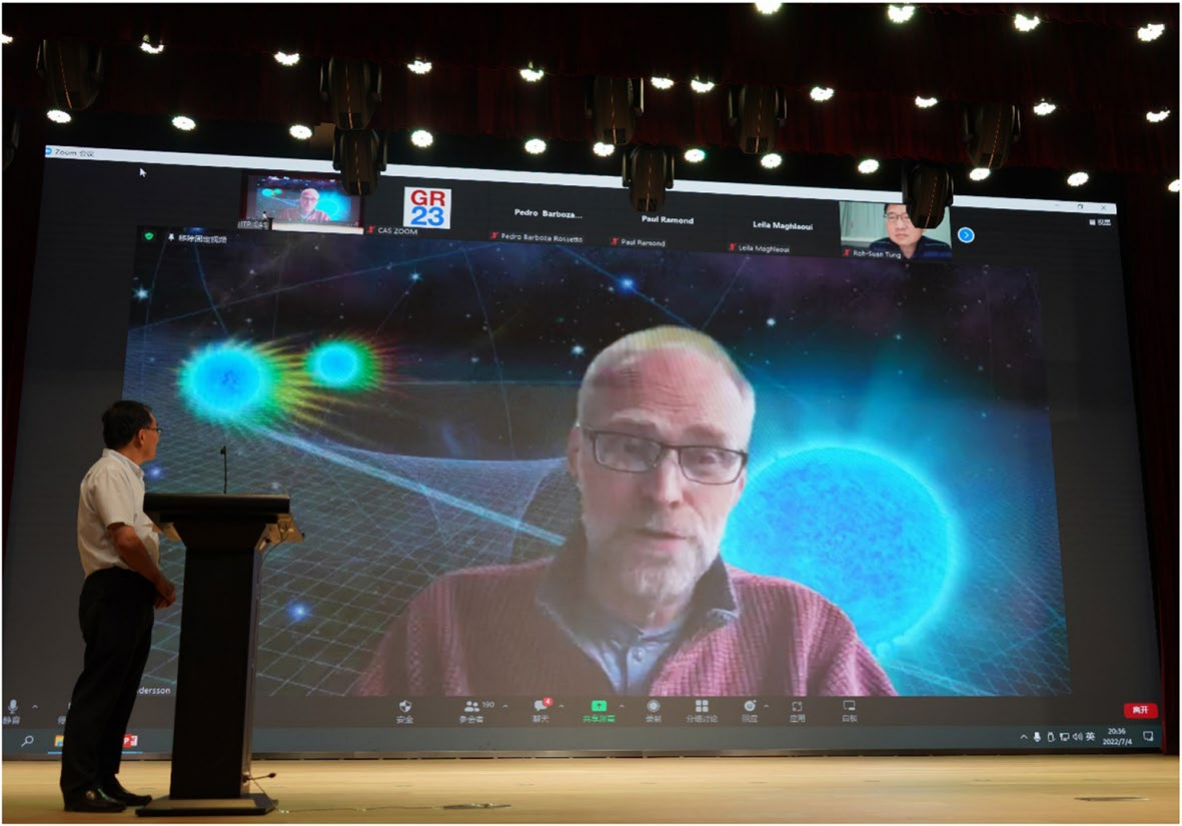
Screenshot of the GR23 opening ceremony

**Fig. 2:**
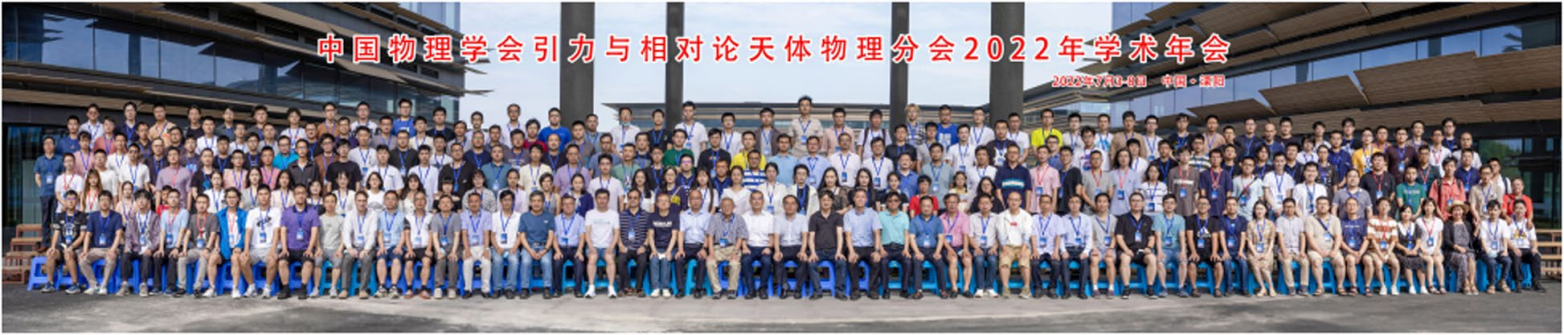
Group photo of onsite participants

Documents pertaining to GR23 (Figs. 1 and 2), including presentation slides and videos, are available on the GR23 website, http://gr23beijing.com/.

## Report on the 48th AAPPS Video Council Meeting by AAPPS

The 48th Council Meeting of the Association of Asia Pacific Physical Societies (AAPPS) was held online from 4:30 p.m. to 6:48 p.m. (UTC+9hr) on May 26, 2022, via a Zoom session hosted by the Asia Pacific Center for Theoretical Physics (APCTP). The participants were Jun'ichi Yokoyama (president), Hyoung Joon Choi (vice president), Nobuko Naka (secretary), Keun-Young Kim (treasurer), Gui-Lu Long (ex officio member as a former president), and council members Tao Xiang (the Chinese Physical Society, Beijing), Xiu-dong Sun (the Chinese Physical Society, Beijing), Ruiqin Zhang (the Physical Society of Hong Kong), Mio Murao (the Physical Society of Japan (JPS)), Akira Yamada (the Japan Society of Applied Physics (JSAP)), Woo-Sung Jung (the Korean Physical Society (KPS)), Kurunathan Ratnavelu (Malaysian Institute of Physics), Rajdeep Singh Rawat (Institute of Physics Singapore), Fu-Jen Kao (the Physical Society located in Taipei), and Nguyen Quang Liem (Vietnam Physical Society). Present as observers were Anisa Qamar (Pakistan Physical Society), Yunkyu Bang (president of APCTP), Jae-Hyung Jeon (executive director of APCTP), Mirim Lee (AAPPS liaison staff member), and Dayoung Yang (AAPPS liaison and editorial staff member). Council members Jodie Bradby (Australian Institute of Physics (AIP)) and Meng-Fan Luo (the Physical Society located in Taipei) were absent and Ying-Jer Kao (the Physical Society located in Taipei) was present as a proxy on behalf of Luo.

(1) Secretary Naka reported the presence of 13 council members (including the proxy) out of 17 council members, and the quorum was declared as fulfilled. [Note: the number of council members present during the 48th Council Meeting changed from 13 to 16.] She informed that the minutes of the 47th Council Meeting were circulated and already published in the AAPPS Bulletin.

(2) President Yokoyama opened the 48th Council Meeting and welcomed the participants. The agenda was adopted as prepared by the president.

(3) Yokoyama explained that some months ago there was an application for membership to AAPPS by the Pakistan Physical Society and introduced Anisa Qamar, the immediate past president of the Pakistan Physical Society. As a representative of the society, Qamar explained the history and current status of the Pakistan Physical Society and their desire to join AAPPS.

Prof. Abdus Salam arranged the 1974 International Nathiagali Summer College, and at this event, Pakistani physicists decided to establish a formal professional body. In 1986, the establishment of a national society of physics was proposed at the National Symposium on Frontiers in Physics. In March 1990, the government of Pakistan formally acknowledged the Pakistan Physical Society. The main objectives of the society are to promote and advance physics, to acknowledge and support excellent researchers and teachers of physics, and to foster linkages between Pakistan and societies/institutions worldwide. Major activities of the society include the organization of conferences and workshops through five local chapters, conducting of executive council and general-body meetings, and awarding prizes. The society offers four categories of membership, as a fellow, honorary fellow, member, or an associate member. A graduate student, holding at least a BS degree or equivalent, is eligible to apply to become a member. Subscription rates are categorized into annual and lifetime fees. Funds of the society are derived from membership fees, grants from the government or international bodies, and donations. The Pakistan Physical Society has, at present, an executive council and five divisions (plasma physics, high-energy physics, condensed matter physics, atomic and molecular optical physics, and women in physics).

Yokoyama asked if the government of Pakistan automatically renews funds every year. Qamar answered that the society applies for a grant every year because at the governmental level, Pakistan’s financial situation is sometimes unstable. Last year, the society made amendments of the constitution and introduced field divisions. The joint sector of all divisions has just started. Qamar was the first female president in the society. She expects that there will be a great deal of progress in different fields in the coming years. The society has organized the first regional e-conference in January 2022 in connection with the centennial celebration of the International Union of Pure and Applied Physics (IUPAP), which was attended by 10–11 eminent speakers and approximately 200 participants.

Kurunathan Ratnavelu commented that a treasurer position is missing in the executive council of the Pakistan Physical Society. Qamar answered that the society does not have a governmental fund yet and they will appoint a treasurer when it becomes necessary. Ratnavelu wondered if approval by the government will be needed once again after significant amendments of the constitution are made. Qamar responded that it will not be needed as the amendments would change only minimal aspects of the constitution.

Yunkyu Bang, the president of APCTP, stated that Prof. Abdus Salam, who had initiated the foundation of the Pakistan Physical Society, also played an important role in the International Centre for Theoretical Physics (ICTP). ICTP and APCTP share the same mission of promoting theoretical physics. APCTP has 17 member countries covering almost all countries in the Asia Pacific region but he noted that a very important society is still missing. Bang invited the Pakistan Physical Society to join APCTP. Qamar showed interest and promised to conduct further discussions.

After Qamar left the meeting, Yokoyama asked whether the council members approve of the Pakistan Physical Society becoming the 20th member of AAPPS. Woo-Sung Jung pointed out that the decision should be made via vote. In the vote, all 15 council members present agreed and the application by the Pakistan Physical Society was unanimously approved.

(4) Vice President Choi reported on the status of preparations for APPC15. The deadline for abstract submission has been extended to May 31st. The number of confirmed invited speakers is 360, excluding those in the Division of Astrophysics, Cosmology and Gravitation (DACG), the Division of Nuclear Physics, and the Division of Plasma Physics (DPP). Already 250 abstracts were submitted from invited speakers (110 are to be reminded). The original plan of having 400 invited speakers will be reached after including speakers from the three listed divisions. At present, the total number of contributed and poster presentations is 140, which corresponds to only 25% of the original plan of 400 contributed talks plus 200 poster presentations. Choi expects more than 90 additional abstracts from DPP. He stated that at APPC15, contributed talks by graduate students as well as by researchers and professors are encouraged. Altogether, we may have approximately 600 abstracts, which is less than the original goal of 1,000 abstracts. Choi would consider it to be a success if we reach 800 abstracts at APPC15.

Registration was planned to start on May 25th but was delayed for a couple of days due to technical issues. The registration fee for middle- and high-school teachers and retired professors will be exempted following the policy of KPS. It should be noted that the exemption is not automatic and requires contact to the secretariat by email. Rajdeep Singh Rawat asked if the exemption is only applied to Korean participants. Choi answered that it is not restricted to Koreans. The temporal list of the invited speakers and titles of submitted abstracts were shown. The opening time of APPC15 will be at 9 am. One room will be prepared for a hybrid session on Sunday and Monday, in which the council meeting (CM), ordinary general meeting (OGM), opening ceremony, and the special sessions, including the Global Physics Summit and the Role of Physics in the Green Economy, will be held.

KPS will provide hotel rooms for those who will come for the CM/OGM. The housing and meals for three nights and four days, starting from Saturday, will be supported. Inside Korea, only two rules regarding COVID-19 remain: masks must be worn indoors and one must self-isolate at home for seven days after testing positive for omicron. If you pass the COVID test when you enter Korea, you can go out of the airport without quarantine. It is expected that the Korean government will eventually announce the lifting of all COVID-19 restrictions and that COVID-19 infections will be treated similarly to an ordinary cold. Airports in Korea are not as convenient to use as compared to pre-pandemic times, but the COVID-19 infection rates are presently low inside Korea. Yokoyama asked if there is a vaccination requirement for travelers entering into Korea. Bang mentioned that foreigners need a certificate of vaccination from their own country. One of the difficulties for participants may be flight costs, which are becoming quite high. The presidents of member societies are invited to participate in the Global Physics Summit. They can participate in person or virtually. Yokoyama confirmed that the onsite venue is at Yonsei University. Yokoyama stated that arriving in Korea is one thing but returning to home countries could cause another problem. When returning to Japan, travelers need to take a PCR test no earlier than three days before the returning flight. Rawat stated that returning to Singapore is even simpler, as there is no need for a PCR test. Rawat thanked Choi for his support to the council members.

Rawat asked Choi if they provide any registration-fee waiver or even partial waiver to the participants with financial difficulties. Choi responded that, although he recognizes the need and difficulties, it is hard to waive the registration fee. Choi added that speakers had to travel previously for APPC14 held in Malaysia, and the support provided was usually limited to their travel. Ratnavelu explained that at APPC14, such participants paid the registration fee for the commitment and then we covered the airfare. However, this time, the only expense is the registration fee. Choi concluded that simply waiving the registration fee is not easy. A significant expense arises as we have more than 10 parallel sessions for five days. Regardless, the registration fee of APPC15 is lower than that for the online participant of the March Meeting of the American Physical Society (APS), which was planned in a hybrid format.

Tao Xiang asked if the receipt of 200 more abstracts is only expected for poster presentations. Choi clarified that both poster presentations and contributed talks (with 300 more slots compared to the original plan) are expected. Yokoyama wondered if the participants prefer the poster format. Choi responded that some students, who are not confident in English, might prefer the poster presentation. Rawat asked if prerecorded presentations are advised for participants who are not comfortable with live oral contributions or have an Internet connection issue. In some conferences, a question-and-answer time is designated following the playback of each prerecorded presentation. Choi stated that live talks are encouraged and a recorded presentation will be accepted as an exception when the speaker absolutely needs to do so. Meanwhile, all poster presenters can submit recorded explanations. The poster session will be held in a metaverse platform where participants appear as avatars. The same poster session room was used at the KPS Spring Meeting, which was convenient and interesting for participants but quite expensive.

Yokoyama asked how many plenary speakers were fixed. Choi answered that 11 speakers out of 14 planned were fixed. He added that three or more additional plenary speakers can be included in the session by changing the last parallel session. He hopes that member societies of AAPPS further encourage submissions for contributed talks and posters.

(5) Yokoyama stated that he received one proposal for the venue of APPC16, which was from the Chinese Physical Society, Beijing.

As a brief introduction to the Chinese Physical Society, Beijing, founded in 1932, Xiang explained that the membership is now 40,000 and there are eight working groups and 32 committees. The society organizes approximately 100 conferences and meetings annually. The executive board comprises the president and six vice presidents. The annual Fall Meeting, held two years ago, was attended by more than 5,000 participants.

Subsequently, Xiang introduced the proposal from the Chinese Physical Society, Beijing, to host APPC16 at the China National Convention Center in Beijing in the autumn of 2025. He explained that the convention center and the hotel are located at a very convenient place within the Beijing Olympic Green and right next to the Bird’s Nest. 100 meeting rooms and advanced audio-visual facilities are available in the convention center. A total of 5000 rooms can be provided at the hotels. The preliminary conference dates are October 7–11, 2025. The budget plan consists of registration fees, funds from the municipal and central governments, on-site exhibiting companies, and publishers. As a point of reference, the Chinese Physical Society, Beijing, also successfully organized APPC11 in Shanghai. Xiang hopes that COVID-19 will not be a serious problem for the conference in 2025 and would work under the assumption that the conference will be held in an in-person format. To summarize, the Chinese Physical Society, Beijing, is well-positioned to host APPC16.

Following the explanation by Xiang, Fu-Jen Kao proposed holding the conference in 2024. He stated that considering the recent progress of AAPPS, including increasing regional exchange activities, holding APPC every two years is worth considering. Xiang responded that the tradition has been to hold APPC every three years but that he passes the decision to AAPPS. Gui-Lu Long reminded that the former council members had previously discussed this issue, but the conclusion was to hold APPC every three years in conjunction with the council term and OGM. Kao commented that APPC does not have to synchronize with the council term. Yokoyama stated that another option is to have it every year, by simply associating APPC with a local society meeting or a division meeting. Yokoyama added that unlike the former time, now we have four divisions, each of which organizes its annual meetings, and the CN Yang Award was changed from every three years to a yearly award. Bang stated that although he is not qualified to say anything about this issue as an observer, APCTP made remarkable progress in every direction these years and we should take this opportunity for the further rapid growth of our community, as an equal counterpart to the European Physical Society (EPS) and APS. He supported Kao’s suggestion of the interval of every two years as a timely change.

Yokoyama mentioned that it depends largely on how many member societies show interest in hosting the conference. Long expressed the same concern. Rawat suggested talking with different societies and people, as both leadership and a great deal of work are required to hold a conference. Long stated that we will lower the target number of participants from 1000 to 800. Kao pointed out that an annual meeting of a local society helps to increase the target. Long suggested holding APPC every three years, while inviting AAPPS scholars to join the AAPPS sessions included in a national conference in the other two years. Yokoyama agreed that some of the divisions may organize such a joint meeting and it is a good way to start holding more frequent APPCs. Choi stated that in order to have APPC more frequently, the preparations should be done more systematically, e.g., by utilizing the same website and contact network as used for annual meetings organized by local societies. To reduce labor and costs, Choi pointed out the need for a committee to handle these matters for APPC.

Kao proposed setting up an APPC working group (WG) under AAPPS. If we have a long-term view of increased activities in the Asia Pacific region, then it will be good to have an APPC WG whose members do not change every year. Choi stated that it will be helpful when we have more divisions in the near future. Rawat supported the suggestion of setting the APPC WG as a subcommittee, just like the CN Yang Award committee under AAPPS. Yokoyama suggested proposing the new WG at a council meeting in August, so that more member societies can participate in the organization of APPC.

Considering the situation with COVID-19 and current travel restrictions, Xiang suggested holding the next APPC in 2025, with the anticipation that international travel will become more normal. In the future, if more societies are interested in hosting APPC, then we can consider having it every two years. Choi supported the proposal by Xiang and added that the actual preparations for APPC will start in 2024. Had we planned to hold APPC16 in 2024, then there would have been more uncertainty regarding the evolving state of the COVID-19 pandemic, as preparations would need to begin in 2023.

When the call for applications was made, Yokoyama asked if a membership fee could be put on top of the registration fee. Rawat commented that it depends on local laws. Long explained that a local account is difficult to open in China. Xiang stated that the money potentially derived from membership fees would be a small amount and it would be better to get support from industries and from publishers. Yokoyama also asked if there is any possibility to associate with a local event. Xiang explained that the annual meeting of the Chinese Physical Society, Beijing, would not be held in Beijing during that time frame and that APPC will be an independent meeting. Yokoyama added that the size of APPC is much smaller than that of the annual meetings of the society, and as such, association with a local event would not be appropriate.

Yokoyama concluded that we shall endorse the Chinese Physical Society, Beijing, to host APPC16 in Beijing in 2025, and that we shall consider establishing a WG to discuss whether future APPCs should be held in shorter intervals. These proposals were unanimously agreed upon by the council members.

(6) Rawat, the chair of the selection committee of the CN Yang Award 2022, reported on the current status of the selection process. Rawat expressed his thanks to Dayoung Yang for her exceptionally good work. We have received 31 nominations from nine different fields and the preselection process has been completed. The final selection stage is to set up a meeting in the coming week or so. The timeline is slightly behind the schedule but conducting the final selection in June should be fine.

(7) Yokoyama reported on the progress regarding the preparation of the code of conduct for AAPPS. He has collected the respective codes of conduct from various societies and associations, such as JPS, JSAP, AIP, KPS (only in Korean, but President Noh provided an English version of the publication code), EPS, and APS. The rules of endorsement, prepared by former President Long, could be used as a part of the code of conduct for AAPPS. Yokoyama asked the council members to send him the code of conduct of their respective member societies if an English version exists. After compiling the collected codes with the members of the code-of-conduct WG, a draft will be proposed in a future council meeting.

(8) Yokoyama reported on the cosponsoring of conferences.

The Thai Physics Society previously asked for the nomination of plenary speakers for the Siam Physics Congress (SPC2022) in Thailand, to be held in June. As the focus of the conference is carbon neutrality, which is applied physics rather than fundamental physics, Yokoyama asked Akira Yamada of JSAP for a recommendation. Yamada kindly found two plenary speakers. The Thai Physics Society also asked AAPPS for financial support. Although we usually do not support such a conference, Yokoyama considered this event to be a worthy opportunity to use a part of $5000 USD, which was provided by JSAP on top of their membership fee. Yokoyama discussed with the vice president, treasurer, and secretary and a contribution of $1500 USD was allocated.

We have already provided $2000 USD of financial support to the Nepal Physical Society for an international conference (ICFP2022), which virtually refills the unpaid membership fees. The Nepal Physical Society asked for speakers to be nominated, and Long, Yokoyama, and Choi attended the conference as plenary speakers.

(9) Naka reported on the status regarding the induction of the next group of council members. We have so far received 12 candidates from nine member societies. She will make a slate soon after the nomination deadline on May 31st and circulate it to the presidents of the member societies for secondment. She pointed out a few typos in the bylaws and constitution, which should be discussed at the OGM in August.

(10) Long briefly explained the current status of the AAPPS Bulletin (AB). He mentioned the editorial board structure and the continued cooperation with Springer Nature and the improved citations of AB. There were 115 total citations in 2021, which was a remarkable improvement as compared to previous years. Articles in high-energy physics, particle physics, plasma physics, and bioscience contributed significantly. There were a total of 18 citations for 23 articles published in 2021, which means an estimated impact factor of 5, and there was a significant jump from 2020 to 2021 in terms of the quality of the papers.

A problem lies in the small number of publications. Presently, a large percentage of the article publishing charge (APC) has been paid for by APCTP. If we could have AB included in the Web of Science and Scopus, Long posits that there would then be more free submissions and then AB could run self-sustainably by the APC collected from the authors. He asked Mio Murao, an expert on quantum information, to contribute and for Choi to make a call to APPC15 participants to request the submission of their best articles to AB.

News and Views are published as one item, electronically, every two months, on the Springer Nature website. The articles are separated in the printed version with a table of contents to allow the readers to access them more easily. This year, four or five original research papers were accepted. A few days ago, AB received an email from EPS asking to publish one of the papers in AB as an article in EPS Newsletters. This shows that AB is getting noticed by EPS. Yokoyama mentioned that AB previously published one article from EPS and EPS published one article from AB. Regarding the editorial board members, Long asked council members for recommendations and, in particular, for recommendations for individuals with backgrounds in applied physics. The senior editors come from member societies, and editors are appointed on an expertise basis. Yokoyama expressed his gratitude to Bang for APCTP’s financial support.

(11) Treasurer K.-Y. Kim gave a brief report on the financial status of AAPPS. The balance is 85,873,176 KRW or $71,560 USD, in addition to the Leo Koguan Foundation’s $36,500 USD (with $10,000 USD excluded for AB). The only expense last year was the establishment fee for the Division of Condensed Matter Physics and up to now, this year’s only expense was the support of $1500 USD for SPC2022 held in Thailand. The account statements include membership fees (bank transfers from Malaysia, the Philippines, and Vietnam are outstanding [*]), AB contributions, and support from APCTP of $468,360 USD. DACG has been supporting and will continue to support the domain fee of $113 USD until 2025. [*Note: payments by the Malaysian Institute of Physics, the Physics Society of the Philippines, and the Vietnam Physical Society were subsequently confirmed.]

(12) Yokoyama announced that the next council meeting will be organized online sometime in late July or early August 2022. The main agenda of the OGM in August will be to report on our activities of the last 2.5 years. Long asked Choi to send invitations and hotel information to the council members.

## Report on the 49th AAPPS Video Council Meeting by AAPPS

The 49th Council Meeting of the Association of Asia Pacific Physical Societies (AAPPS) was held online from 3:00 p.m. to 5:38 p.m. (UTC+9hr) on July 28, 2022, via a Zoom session hosted by the Asia Pacific Center for Theoretical Physics (APCTP). The participants were Jun'ichi Yokoyama (president), Hyoung Joon Choi (vice president), Nobuko Naka (secretary), Keun-Young Kim (treasurer), Gui-Lu Long (ex officio member as a former president), and council members Xiu-dong Sun (the Chinese Physical Society, Beijing), Ruiqin Zhang (the Physical Society of Hong Kong), Mio Murao (the Physical Society of Japan (JPS)), Akira Yamada (the Japan Society of Applied Physics (JSAP)), Woo-Sung Jung (the Korean Physical Society (KPS)), Rajdeep Singh Rawat (Institute of Physics Singapore), and Nguyen Quang Liem (Vietnam Physical Society). Present as observers were Yunkyu Bang (president of APCTP) and Dayoung Yang (AAPPS liaison and editorial staff member). Council members Jodie Bradby (Australian Institute of Physics (AIP)), Tao Xiang (the Chinese Physical Society, Beijing), Kurunathan Ratnavelu (Malaysian Institute of Physics), Fu-Jen Kao (the Physical Society located in Taipei), and Meng-Fan Luo (the Physical Society located in Taipei) were absent. Hai-Qing Lin (the Chinese Physical Society, Beijing) and Ying-Jer Kao (the Physical Society located in Taipei) were present as proxies on behalf of Xiang and Luo, respectively.

(1) Secretary Naka reported the presence of 14 council members (including the two proxies) out of 17 council members, and the quorum was declared as fulfilled. President Yokoyama informed that the minutes of the 48th Council Meeting were circulated by e-mail.

(2) Yokoyama opened the 49th Council Meeting and welcomed the participants. He introduced Hai-Qing Lin and Ying-Jer Kao, who were present as proxies. The agenda was adopted as prepared by the president.

(3) Vice President Choi reported on the status of preparations for APPC15. On the top website of APPC15, three logos of the 70th anniversary of the KPS, the International Year of Basic Sciences for Sustainable Development, and the Centennial of the International Union of Pure and Applied Physics (IUPAP) are posted. On the morning of Monday, August 22, we have two speakers in the plenary session followed by the parallel sessions, which include the special session on the Global Physics Summit. In the afternoon, there are two parallel sessions (with a break), including the special session on the Role of Physics in the Green Economy (without a break) as well as the poster session. The ceremony of the CN Yang Award will be held from 1:30 p.m. to 2:00 p.m. in a hybrid format, right before the afternoon parallel sessions. Three winners of the CN Yang Award will deliver talks in the fully online session from 4:00 p.m. to 5:30 p.m.

The schedule of contributed talks was already announced to all speakers. The schedule of the 14 plenary speakers is not fully fixed yet. One change will be made on the slot of one plenary speaker from Europe, whose time zone is not good. The alternative slot will be the period of the break from 3:30 p.m. on Tuesday. Then, there will be room to have one more plenary speaker. Choi stated that we currently have no plenary speaker from the particle physics field, and recommendation is welcome. So far, slightly more than 1000 abstracts, including 510 invited talks, 330 contributed talks, and 190 posters, have been accepted. This means that we reached the initial goal of making APPC15 successful.

The deadline for the early-bird registration was July 15 and over. The regular registration will start on August 1 and continue until the end of the conference. About 800 people have so far made the registration. The withdrawal rate seems to be lower than the previous experience of 10% for other conferences. The local organizing committee decided to waive the registration fees for five participants from Nepal and another five from Myanmar. The registration fee for retired professors and high-school and mid-school teachers is exempted.

On Sunday, August 21, the Ordinary General Meeting (OGM) will be held in the faculty meeting room of the Physics Department at Yonsei University, Seoul. The room was recently renovated with equipment for hybrid meetings. Among presidents of member societies and council members, five people will attend the meetings in person, while others will participate virtually.

Yokoyama asked about the distribution of member countries and regions. Choi answered that 40% of the abstracts were submitted from inside Korea. JPS, JSAP, the Chinese Physical Society, Beijing, and the Physical Society located in Taipei made the next largest contributions for another 40%. Some significant numbers of participants choose their society as the American Physical Society (APS). Choi added that probably not all of them live in the United States (US), but are APS members in other countries/regions. Many abstract authors from India and Pakistan requested a full or partial waiver of the registration fee. The waiver was limited to participants from Nepal and Myanmar, which are included in the UN list of least developed countries. Choi explained that he plans to discuss with Gui-Lu Long shortly to prepare an editor session.

Yokoyama explained that AAPPS and AAPPS Bulletin had received an invitation from the KPS to participate in the virtual booth on the metaverse platform for the exhibition and poster sessions. Choi explained that the booths for AAPPS, AAPPS Bulletin, and APCTP are provided free of charge, thanks to the support of the KPS. When you access the platform website, you will be asked for your email address. After clicking the confirmation email, you can enter the metaverse after choosing your avatar, clothes, and the head. In the poster session, the presenters can discuss using the shared presentation materials with the participants. At the booths, we will provide links to materials such as viewgraphs, videos, and websites of AAPPS and AAPPS Bulletin. Yokoyama suggested adding power-point presentations to introduce the activities of AAPPS, prepared by the president.

Yokoyama suggested making recordings of plenary sessions for later service, for example, for member societies that cannot send many participants to APPCs. Choi responded that we do not have any plan to record scientific sessions but may record the first hybrid session for the opening, the first plenary session, and the special sessions. Rawat conveyed a question if all the presentations will be given in parallel Zoom sessions. Choi answered that we have a special Internet connection for APPC15 from a cable company, independent of the Yonsei University’s Internet line, which enables all sessions to progress with live talks and live Q&A. Rawat pointed out that we need permission from all the speakers for recording, and recorded videos should be set only playable and not downloadable. Choi responded that we cannot hamper recording by the audience as recording videos is one of the standard tools in the Windows operational system. Rawat also commented that students feeling uncomfortable with languages sometimes prefer pre-recorded presentations. Choi answered he knows it but encourages students to deliver a live presentation for educational purposes. If some speakers request recorded presentations, we will consider it positively but will not announce it. We should be careful handling large-size recordings to be virus-free.

Yokoyama asked about the number of parallel sessions. Choi responded that there are 13 subjects on top of special sessions and the session for Women-in-Physics. The average number of sessions running simultaneously is 17. The technical aspects of the sessions are controlled by secretaries, and the session chairs handle academic or scientific parts only. Invitations to session chairs will be sent in the next week.

(4) The plan for Sunday, August 21, the day before the start of APPC15, was discussed. Normally, we have a council meeting, OGM, new council meeting, and extended council meeting. The extended council meeting will be attended by outgoing and incoming council members. Considering the time difference of the member countries/regions, the starting time and duration of the first council meeting were set at 10:00 a.m. and two hours, respectively. After a lunch break, the OGM will start at 1:00 p.m., whose agenda includes a report on the AAPPS activities of the past 2.5 years and the election of the new council members. The previous election was easy because we had the same number of candidates and available positions. This time, we have more candidates than the number of seats, and the election should be done in a hybrid manner. The duration of OGM is estimated to be two hours.

Woo-Sung Jung explained the agenda of the previous OGM, prepared by the AAPPS headquarters. He confirmed that the vote is confidential and that the introduction of participants took the longest time among the agenda items. Yokoyama stated that, unlike other APPCs, we have APPC15 in the middle time of the year while our term continues until December. He commented that all the forthcoming council meeting(s) after August would be a kind of extended one participated by both outgoing and incoming council members. He considers that it is better to have some term overlaps, as the new members will become familiar with AAPPS activities. Long stated that it is up to the president to decide whether to invite new council members. The term of the new secretary will start in January 2023, and the new president has time to consider the appointment.

Yokoyama stated that one hour would be enough for the new council meeting (not observed by current council members), while Jung pointed out that it took more than one hour in 2016. The time of the new council meeting was set at 3:30 p.m.–4:30 p.m. The ending time of the new council meeting is tentative, and the current council members will wait in another Zoom room for the new council members to join the extended council meeting. The extended council meeting was scheduled (tentatively) from 4:30 p.m. to 6:00 p.m. Choi confirmed to prepare four different Zoom meeting rooms for Sunday.

(5) At the time of the previous OGM, the president of the KPS mentioned that we should have more members from more different countries and regions in the council. Yokoyama asked about opinions on the possibility of expanding the size of the council, considering the increasing number of member societies of AAPPS. Yokoyama explained that this issue involves an amendment of the Constitution, which requires approval at a General Meeting. He had thought that such an important issue should be discussed well at in-person meetings rather than online meetings forced during the COVID-19 time. Choi pointed out that we would need to inform the member societies well in advance, e.g., six months before to discuss. Long expressed his opinion that it is the right time to discuss this issue. Rawat and Ruiqin Zhang supported proposing an amendment of the Constitution at the OGM in August 2022. Choi also agreed and wondered if what number would be appropriate. Jung suggested just raising the point and not immediately deciding the number. Mio Murao asked about the possible disadvantage when the council is expanded and if we expect that some of the agreements will become difficult to reach, for example. No concrete cons were pointed out.

Meanwhile, President Yokoyama received a written proposal to increase the number of council members from Ying-Jer Kao as the president of the Physical Society located in Taipei. Yokoyama asked for opinions from council members, and the proposal was seconded by all the present council members.

Subsequently, the number to be increased was discussed. Murao asked about what was the number of member societies when the number of council members was defined as 15. Long introduced that the members at the time of the AAPPS foundation in 1989 were 15 societies plus the ASEAN Institute of Physics and South East Asia Theoretical Physics Association. The latter two are presently no active members. Jung stated that the number of member societies was 16 in 1997 and has increased by four as of today. He proposed 20 council members for the amendment. Kao wondered if an odd number would be more favored for voting. Yokoyama explained that the ex officio and secretary, who are voting members, are not included in the current number of 15 and a treasurer, if they were not elected, is a non-voting member of the council. The 20 means the upper limit, and no change will be proposed for the lower limit of 9. Rawat asked if the quorum is defined by an absolute number or in percentage. Naka confirmed that it is defined as two-thirds of the council members, and there is no need for an amendment regarding the quorum. Long added that the maximum number of council members from member societies in one country/region is also defined in percentage.

Yokoyama finally proposed to decide on the tentative upper limit of 20 and to make the final decision with the opinions of member societies at the OGM. The motion was unanimously agreed upon by the council members.

(6) Yokoyama reported on his recent activities as the president. He attended the conference of the Italian Physical Society held online and then made his first international trip after the COVID-19 pandemic to attend the IUPAP Centennial Symposium in Trieste. Yokoyama briefly showed the presentation slide. He explained cooperation with APCTP, whose support is always appreciated, organization of APPCs, support for the regional activities organized by the Nepal Physical Society and the Thai Physics Society, as well as the division activities. Women-in-Physics activities are popular here, and the number of female physicists exceeds that of male physicists in some countries. There is significant diversity among our member societies in terms of size, structure, and stage of development and decline. Statistics about Japan represent no increase in GDP, no inflation, and no increase in our salary over three decades. Thus, Japan has established a stationary state as an environment-friendly society with a sustainable economy. However, the government debt increases more and more, and it will reach some limit beyond which we would not be able to sustain. The budget for science has not increased very much in Japan compared to the year 2000. Meanwhile, China and Korea have significantly increased spending on science, leading to the rapid growth of research on physics in the region.

Yokoyama also introduced activities of some members of the Division of Nuclear Physics (DNP) to support physicists in Myanmar. The country was democratized in 2011 but turned to military rule by the coup d’état in 2021. There was a Campaign for Civil Disobedience against the military government, which led to a serious division between people involved and not involved in the movement. The DNP has been organizing lectures and experimental courses or even inviting researchers to Japan for further education, without discrimination whether those who joined or not joined the Campaign. Yokoyama expressed that he wishes to provide some support to these activities in Myanmar from AAPPS. However, they are not a member society of AAPPS, and it may not be a good idea to spend the AAPPS money. He suggested using the honorarium, which he had received for writing an article published in the AAPPS Bulletin, for donation on behalf of AAPPS. Yunkyu Bang expressed no objection.

After the IUPAP meeting, Yokoyama met the president-elect of the IUPAP, who showed interest on the AAPPS Bulletin and proposed cooperation with IUPAP. In order to meet this request, Yokoyama suggested even strengthening the News and Views section of the AAPPS Bulletin, which is its unique feature. Long welcomed the proposition. Long added that the quality of papers in the AAPPS Bulletin is significantly increasing, and the problem of the small number of articles will be resolved in the future with cooperation with Springer Nature.

(7) Just before the COVID-19 pandemic, Yokoyama attended the annual meeting of the Physical Society located in Taipei and introduced the activities of AAPPS at the council meeting of the society. He further proposed to start a joint award between AAPPS and member societies for young researchers. The council members of the society agreed to establish one. AAPPS council also agreed to try it as a joint pilot program of AAPPS and the society. The first-year prizes were given to three researchers who delivered excellent talks at the annual meeting and had relations outside the society region after one year’s postponement due to the pandemic. The winners received gold medal plates from AAPPS, which were donated by the AAPPS president, and monetary prizes from the Physical Society located in Taipei. The cash prize was approximately 100 USD as a token. The current president, Kao, intends to continue this program.

Yokoyama recently reported on this pilot program at the AAPPS committee meeting inside JPS. They were positive about establishing a new joint award for AAPPS and JPS. The idea of JPS is that they will select winners of the AAPPS-JPS Award among nominees for the CN Yang Award who were recommended by JPS divisions but not the final recipient of the CN Yang Award. JPS can nominate up to 19 candidates for the CN Yang Award, and usually, not all the divisions make nominations. The nominees are all excellent, and JPS will consider them as candidates for the AAPPS-JPS Award. Yokoyama explained that, by doing so, we could encourage talented young researchers and increase the visibility of AAPPS more prominently in member societies. He learned at the IUPAP meeting that the Young Scientist Prize (currently renamed the Early Career Scientist Prize) was established for the purpose of making young researchers know about the IUPAP. A draft of guidelines for the joint award with member societies was prepared and explained by Yokoyama.

Long expressed his support for this scheme. He added a comment that we should be careful about the naming of the joint prize not to be confused with the CN Yang Award. Long also suggested raising the profile of the award to make AAPPS much more visible. Yokoyama responded that it would be unfortunately more difficult to achieve. Rawat stated that he considers formulating the joint awards a fantastic idea. He wondered if a certificate will be prepared by the local society. Long suggested including the logo of AAPPS in the certificate. Kao stated that he would share an exemplary certificate prepared by the Physical Society located in Taipei with the council members for reference. Consequently, the draft of guidelines for joint awards was approved by the council members.

(8) The code of conduct making has been pending for quite a while. At the previous council meeting, Yokoyama reported that he had collected the respective codes of conduct from various societies. A draft of the Code of Conduct of AAPPS was presented by Yokoyama, and he asked for opinions and comments by the time of the next council meeting when it will be finalized.

(9) Thanks to the generous support of APCTP, the website of AAPPS has been under complete renovation. Yokoyama asked council members to give any inputs and opinions. Choi suggested carefully confirming the names of member societies.

(10) Yokoyama explained that the Physics Society of Iran wishes to join AAPPS. Following the convention, he asked the society to fill out the questionnaire form. The form says that the establishment year is 1963, while the website says that the creation was in 1932, which means the society is as old as the Chinese Physical Society, Beijing. The society has 2500 regular members and more than 8000 student members. The society organizes annual meetings and has a division structure, publishes the Iranian Journal of Physics Research, and awards a number of prizes. They wish to join as an associate member till someday when the political sanction by the US is lifted, and they can send money abroad.

Rawat commented that the level of physics research in Iran is high, and Singapore has a substantial number of Iranian students. According to his involvement in the International Physics Olympiad (IPhO), the Iranian team always does very well. They are also known internationally as they organized IPhO 2007 in Isfahan with great success. Therefore, Rawat considers it is good to involve them in AAPPS. Bang explained that APCTP previously had a few Iranian postdocs. Because of the US sanction, however, newly coming Iranian researchers cannot open a new bank account in Korea, which made them finally not come. Yokoyama stated that he personally knows many Iranian physicists and has published a collaborated paper with one of them.

Yokoyama summarized that he hopes to make a final decision on whether we should accept them or not after having a presentation by the president of the society at a future council meeting.

(11) Yokoyama explained Clause 7.3 of the Constitution, stating, “Any Member Society which is in arrears in the payment of membership fees shall have no vote at General Meetings.” Previously, not all the member societies gathered at the OGM, and some societies in arrears of payment usually did not come. However, the next OGM will be held in a hybrid format, and some member societies with unpaid fees may attend. Yokoyama asked what we should do, i.e., send them the electronic voting ballot, ask them to finish the payment, or having joint activities with AAPPS to virtually support, or simply follow the Constitution.

Rawat stated that we should follow the clear statement in the Constitution. Long also respects the Constitution and states that member societies with unpaid fees can take part in the activities (including participation in the OGM) but should not have a vote. Long recommended that Naka write a letter notifying Clause 7.3 to those members and give them a chance to make a payment by the time of the OGM.

(12) Rawat conveyed a question about the AAPPS fellowship. He explained that fellowship makes the society more visible, and fees from fellows are funding resources in Singapore. Yokoyama responded that we do have some AAPPS honorary fellows already, but we do not ask them to pay. Long explained that rules to appoint an AAPPS honorary fellow have been formulated by Prof. Seunghwan Kim. Only very limited scholars, such as Nobel Prize winners and past presidents who made a significant contribution to AAPPS, have been appointed so far. Rawat suggested including the list of AAPPS honorary fellows on the new website.

Yokoyama announced that the next council meeting would be held on August 21, 2022, and closed the meeting.

